# MMW Radar-Based Technologies in Autonomous Driving: A Review

**DOI:** 10.3390/s20247283

**Published:** 2020-12-18

**Authors:** Taohua Zhou, Mengmeng Yang, Kun Jiang, Henry Wong, Diange Yang

**Affiliations:** State Key Laboratory of Automotive Safety and Energy, School of Vehicle and Mobility, Tsinghua University, Beijing 100084, China; zhouth18@mails.tsinghua.edu.cn (T.Z.); yangmm_qh@mail.tsinghua.edu.cn (M.Y.); jiangkun@mail.tsinghua.edu.cn (K.J.); huangqj19@mails.tsinghua.edu.cn (H.W.)

**Keywords:** MMW radar, autonomous driving, environmental perception, self-localization

## Abstract

With the rapid development of automated vehicles (AVs), more and more demands are proposed towards environmental perception. Among the commonly used sensors, MMW radar plays an important role due to its low cost, adaptability In different weather, and motion detection capability. Radar can provide different data types to satisfy requirements for various levels of autonomous driving. The objective of this study is to present an overview of the state-of-the-art radar-based technologies applied In AVs. Although several published research papers focus on MMW Radars for intelligent vehicles, no general survey on deep learning applied In radar data for autonomous vehicles exists. Therefore, we try to provide related survey In this paper. First, we introduce models and representations from millimeter-wave (MMW) radar data. Secondly, we present radar-based applications used on AVs. For low-level automated driving, radar data have been widely used In advanced driving-assistance systems (ADAS). For high-level automated driving, radar data is used In object detection, object tracking, motion prediction, and self-localization. Finally, we discuss the remaining challenges and future development direction of related studies.

## 1. Introduction

At present, the rapid development towards higher-level automated driving is one of the major trends In technology. Autonomous driving is an important direction to improve vehicle performance. Safe, comfortable, and efficient driving can be achieved by using a combination of a variety of different sensors, controllers, actuators, and other devices as well as using a variety of technologies such as environmental perception, high precision self-localization, decision-making, and motion planning. MMW radar, as a common and necessary perceptive sensor on automated vehicles, enables long measuring distance range, low cost, dynamic target detection capacity, and environmental adaptability, which enhances the overall stability, security, and reliability of the vehicle.

Based on the measuring principle and characteristics of millimeter waves, radar perception data has the following advantages compared with other common perceptive sensors such as visual sensors and LIDAR [1]: first, MMW radar has the capability of penetrating fog, smoke, and dust. It has good environmental adaptability to different lighting conditions and weather. Secondly, Long Range Radar (LRR) can detect targets withIn the range of 250 m. This is of great significance to the safe driving of cars. Thirdly, MMW radar can measure targets’ relative velocity (resolution up to 0.1 m/s) according to the Doppler effect, which is very important for motion prediction and driving decision. Due to these characteristics of MMW radar and its low cost, it is an irreplaceable sensor on intelligent vehicles and has been already applied to production cars, especially for advanced driving-assistance systems (ADAS).

**Figure 1:**
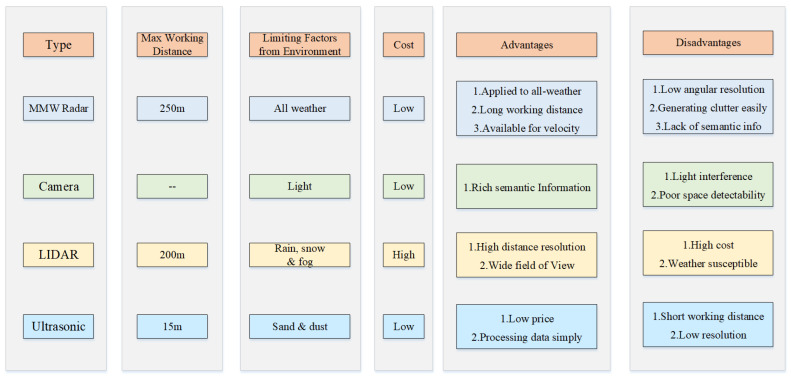
Comparisons of different sensors.

However, MMW radar also has some disadvantages [2,3]: first, the angular resolution of radar is relatively low. To improve the angular resolution, the signal bandwidth needs to be increased, which costs more computing resources accordingly. Second, radar measurements lack semantic information which makes it impossible to fully meet perception requirements In high-level automated driving. Third, clutter cannot be completely filtered out of radar measurements, leading to false detections which are hard to eliminate In the subsequent data processing. More detailed comparisons between MMW radar and other on-board sensors are listed In Figure 1.

As a result of the characteristics listed above, radar-based models for autonomous driving are established. Different automated driving levels require different radar information [4]. For low-level AVs, MMW radar provides object-layer data to perception input which is applied to active safety technologies, such as collision avoidance, lane changing warning, blind spot detection, etc. [5,6]. Among these applications, MMW radar data processing focuses on filtering out clutter to obtaIn stable object trajectory and achieving full coverage of vehicles by avoiding blind areas to reduce driving risks. However, high-level AVs demand much more precise, comprehensive, and robust environment information. Object-layer radar data cannot satisfy corresponding perception demands. Therefore, original point cloud information before clustering and tracking which is called cluster-layer data is used more frequently at high-level automated driving. In these applications, raw point cloud data of single snapshot is used to obtan object dimension [7,8], orientation, motion estimation [9,10], and object category [11,12]. Then, raw radar data accumulated from multiple snapshots is used to build grid maps [13,14]. These representations are used to express dynamic and static environment elements and applied to many applications such as object detection and tracking [8,15,16], environment mapping, and vehicle localization [17,18,19].

Moreover, through multiple sensor fusion with visual sensors and LIDAR, the system can obtaIn a better understanding of driving environment. Driven by the breakthroughs brought by deep learning (DL) techniques, plentiful wonderful deep neural networks (DNNs) are applied to perception tasks. Due to the powerful learning capacity of DL, the performance of related tasks is improved massively. Quite a lot of DL frameworks have been investigated on images and LIDAR data as images and LIDAR point clouds provide abundant data for deep neural network to traIn and validate [20,21,22]. Compared with vision and LIDAR, radar-related DL studies are much less as radar data is relatively sparse. However, although DL techniques based on visual sensors and LIDAR have been developed more adequately, special situations exist where the two sensors cannot work ideally, such as In raIn and snowfall. Therefore, MMW radar can be used as a sensor to robustly observe surrounding information In these situations. In addition, the latest research works for autonomous driving focus increasingly on using radar sensing data to realize fully environmental perception. Some successful DL techniques are applied to radar data. Specific convolutional neural networks (CNNs) and recurrent neural networks (RNNs) are proposed for radar data processing [23,24]. Furthermore, related studies use DNN to improve the fusion performance [25,26]. To satisfy the enormous demands of radar data In deep neural network (DNN) training, new datasets carrying the full autonomous vehicle sensor suite of radar, LiDAR, and visual sensors such as nuScenes are published [27].

There are also several surveys on MMW Radar In autonomous driving which have been published [2,3,28]. They introduced radar perception approaches for autonomous driving from detection and ranging. Compared with these previous reviews, we add the latest developments and give a more sufficient understanding on DL-related research fields.

The maIn contributions of our work can be summarized as three points:

- An organized survey of MMW radar-related models and methods applied In perception tasks such as detection and tracking, mapping, and localization.
- Latest DL frameworks applied on radar data are fully investigated.
- A list of the remaining challenge and future direction which can enhance the useful application of MMW radar In autonomous driving.

The remainder of the paper is organized as follows. Section 2 introduces data models and expressions of MMW radar. Section 3 discusses applications related to MMW radar In autonomous driving. Section 4 provides an overview of future development. Section 5 draws conclusions on current research works.

## 2. Data Models and Representations from MMW Radar

Millimeter-wave radar can provide cluster-layer data and object-layer data. Cluster-layer data provides more abundant information with more noise while object-layer data gives less noisy and sparser data after filtering and tracking. Thus, according to different application demands of AVs, radar data can be used to construct a variety of models to represent environmental information sufficiently. According to the distinction between object motion states, radar-based representations can be divided into dynamic object modeling and static environment modeling. For convenience, we summarize all the radar-based modeling methods In Table 1.

### 2.1. Dynamic Target Modeling

**Figure 2:**
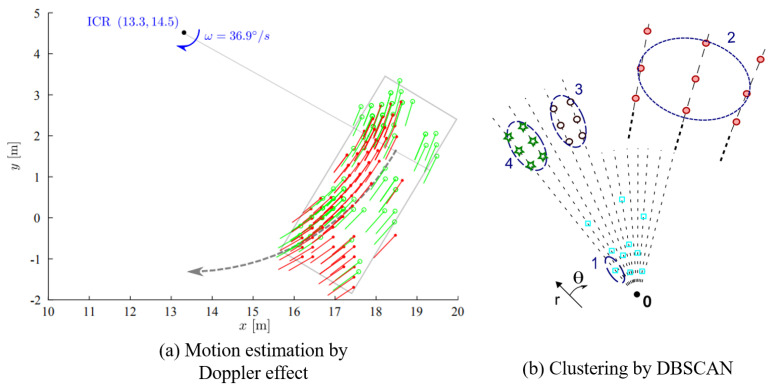
The effect of dynamic modeling based on radar data [9,30].

Original point cloud information of a single snapshot can be used to estimate extended information of a dynamic target. Two main methods are used to acquire object dimension, outline, orientation, and motion state of the whole target. The estimation effect of the two methods is listed In Figure 2.

The first method uses Doppler data of two 77 GHz automotive radar sensors to estimate the velocity profile of an extended object. The outliers which do not belong to the same object are filtered by RANSAC. Then, full 2D-motion state (yaw rate, longitudinal and lateral speed) of an extended dynamic object is acquired. Through velocity profile analysis, related parameters of the instantaneous center of rotating (ICR) $\left(\omega ,x_{0},y_{0}\right)$ are estimated. Then, the target size $\left(w,h\right)$ and target movement are inferred [9,35]. This algorithm performs strongly In real time as the processing time cycle is about 50 ms. It is also very robust as the algorithm is resistant to white noise and systematic variations In the signal. However, when the object cannot be clearly extracted from the data of a single scan, such methods tend to fail.

When multiple radar reflections from one object are measured, a direct scattering approach as well as an extended object estimation method have been specifically used In [10,29].

The second method is using DBSCAN (Density Based Spatial Clustering of Applications with Noise) for original point cloud clustering and estimation of the extended information of targets such as dimension and orientation. DBSCAN is a widely used clustering method to process the original point cloud of MMW radar. As radar point cloud is quite sparse as well as contains vast clutter and nonuniform density, the partitioned clustering method (e.g., K-Means) and hierarchical clustering algorithm are both inapplicable to process radar data. On the other hand, DBSCAN is adaptive to cope with the difficulties listed above. Grid-based DBSCAN algorithm uses r−θ grid modeling to solve the clustering difficulties caused by low angular resolution [7,30]. On this basis, Doppler velocity is added to help improve the clustering effect and adaptive clustering method for tracking is introduced to further enhance algorithm realizability [8]. The methods listed above are suitable for high-resolution radar data. As these algorithms need the radar to detect much more reflection points from one single object to perceive the driving environment precisely. Therefore, a high-resolution radar is strongly demanded. Moreover, dynamic modeling methods have been used to realize extended object tracking combined with Random Finite Sets (RFS) [16] or tracking frameworks which have adapted to extended object tracking [29].

**Figure 3:**
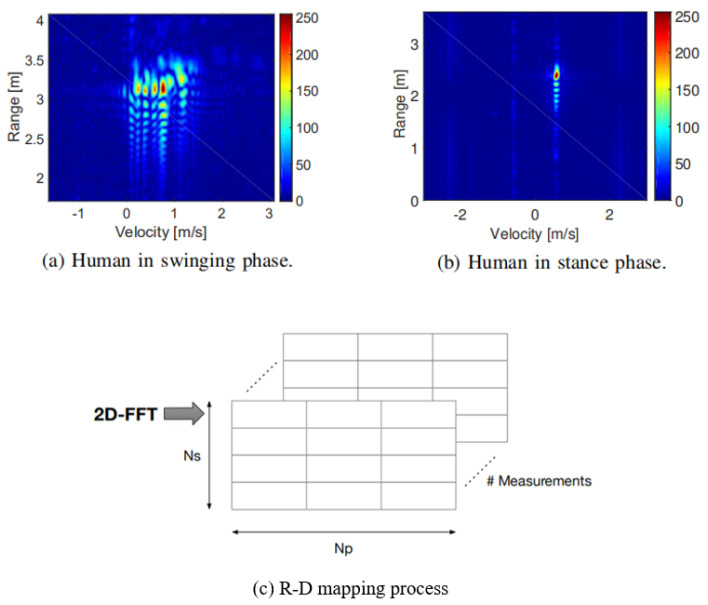
An illustration of R-D Map [12].

In addition, MMW radar detections of dynamic objects hold other available properties. The Micro-Doppler effect refers to frequency modulations centered about the maIn Doppler frequency In the MMW radar signal procedure due to the micro motion of the moving object’s body and limbs, such as rotation and vibration. This is a type of identification, closely associated with the target motion state, which can be used to analyze target movement characteristics for target classification, motion recognition, and pedestrian detection tasks [11]. When MMW radar procedure applies 2D Fast Fourier Transform (FFT) from the reflected radar signals, a joint Range-Doppler (R-D) Map is obtained. The size of R-D Map is related to the range resolution and velocity resolution. Each R-D map contains rich information about the Micro-Doppler effects of dynamic objects. As is displayed In Figure 3, the R-D Maps of human In different phases (swinging vs. stance) reveal quite different features.

### 2.2. Static Environment Modeling

While a vehicle is driving, multiple MMW radar snapshots can be accumulated to build an environment map and realize the representation of static environment. There are two different grid-mapping algorithms based on radar data. One is the occupancy-based grid-mapping, and the other is the amplitude-based grid-mapping [13]. Traditionally, the most widely used method to perform grid-mapping is using an inverse sensor model (ISM) and Bayesian filtering techniques [14].

#### 2.2.1. Occupancy Grid Map (OGM)

Occupancy grid maps represent the probability of each cell being empty or occupied. The cell value of a grid map *m* at $\left(x,y\right)$ is a binary random variable. When it is occupied at time *t*, $m_{x,y}\left(t\right)=1$, otherwise $m_{x,y}\left(t\right)=0$. Radar sensor measurements $Z_{1:t}$ and pose information $X_{1:t}$ are used to estimate the probability of whether a cell is occupied $P\left(m_{x,y}\left(t\right)|Z_{1:t},X_{1:t}\right)$. Meanwhile, the logarithm form can be used to avoid extremely large or small probability values.

(1) $$L\left(m_{x,y}\left(t\right)\right)=log\frac{P\left(m_{x,y}\left(t\right)|Z_{1:t},X_{1:t}\right)}{1-P\left(m_{x,y}\left(t\right)|Z_{1:t},X_{1:t}\right)}$$

#### 2.2.2. Amplitude Grid Map (AGM)

Besides target localization, amplitude grid maps reflect the RCS of radar detections. Since radar amplitude refers to the reflection cross-sectional area value of radar signal, it is related to the reflection attribute of targets and can distinguish metal and non-metal materials. Amplitude grid map cell value $m_{x,y}\left(t\right)$ at position $\left(x,y\right)$ is the weighted mean of all radar observations amplitudes $A_{x,y}\left(k\right)$ of this cell up to time step *t*, $\left(0\leq k\leq t\right)$.

(2) $$m_{x,y}\left(t\right)=\frac{\sum_{k=0}^{t}\left(\left(\frac{1}{r_{x,y}\left(t\right)}\right)A_{x,y}\left(k\right)\right)}{\sum_{k=0}^{t}\left(\frac{1}{r_{x,y}\left(t\right)}\right)}$$

**Figure 4:**
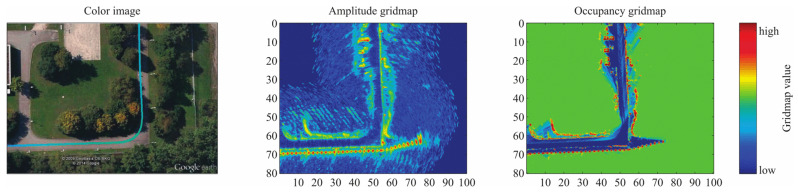
An illustration of automotive radar grid maps [13].

Because of the different modeling approaches, the two grid models hold different qualities. The contour features and position properties of OGMs are usually clearer, while the AGMs can express more characteristics of targets. An illustration of this two different mapping approaches is showed In Figure 4. Suitable grid map types can be selected according to different requirements.

Besides using inverse model and Bayesian framework, Degerman et al. proposed an adaptive gridding algorithm [31]. They extracted signal-to-noise ratio (SNR) with a Swerling model, to give different occupancy probabilities for measurements. They then used a fast trilinear interpolation to update the grid. Besides the methods of building grid maps listed above, new studies try to use deep learning to solve the same problem. They use ground truth from LIDAR and supervised learning to realize occupancy grid-mapping for static obstacles, from radar data on nuScenes [14].

The choice of suitable gridding and mapping solutions from different algorithms is based on different situations. Wen, Z. et al. used quantitative quotas to evaluate the map quality and choose the better one [36].

#### 2.2.3. Free Space

Free space refers to areas where vehicles can pass freely without other traffic participants. The free space is defined by the narrowest distance between the vehicle possible position and the border of the occupied space. Based on radar grid maps describing the static environment, free space can be further determined based on the border recognition algorithm [34]. Compared with LiDAR, occupied objects can be better detected, and a more accurate free space range can be obtained with radar due to its penetrability [33].

**Figure 5:**
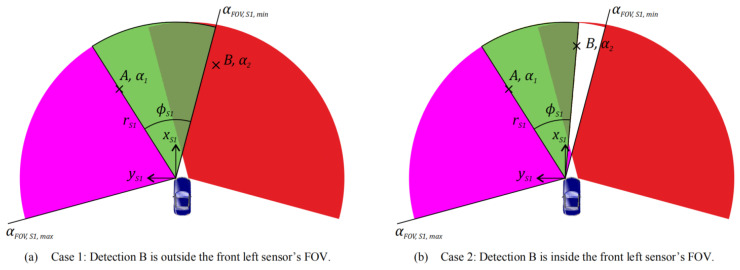
An illustration of free space (pink and green) determined by radar (the front left) [33].

Figure 5 shows the additional free space for the front left sensor In different scenarios. Let A and B be the detection of the front left and front right sensor. Let $\alpha_{1}$ and $\alpha_{2}$ be the smallest azimuth to the front left sensor’s FOV and the highest azimuth to the front right sensor’s FOV. Let $\alpha_{FOV,S1,min}$ be the lower limit of the front left sensor’s FOV with the range $r_{S1}$. In addition, additional free space exists when $\phi_{S1}\geq \phi_{min,S1}$ in the two different cases.

In summary, the establishment of AGMs and OGMs are important representations of static environments from automotive radar data, which can be applied to lane prediction, free space description, parking detection, SLAM, and other autonomous driving tasks [32,37,38]. Compared with LIDAR, the advantages of using radar data In environmental mapping include low cost, high adaptability, and the ability to detect partially occupied objects. The disadvantages are lower resolution and precision of measurement.

### 2.3. Association between Dynamic and Static Environment

The association between static environment map obtained by multi-frame information and dynamic target obtained by single frame information contributes to a more comprehensive and accurate understanding of the actual driving environment. Typically, static environments have been already represented with global grid maps. Therefore, the association between static environments with dynamic targets need to be achieved by relative modeling. There are two methods. The first is to derive free path and extract semantic information through static environment representation, and then express dynamic targets on the free path In the form of point clouds [39]. The second is to construct a local occupancy grid map for dynamic targets. The correlation between the local grid map and static global grid map can be realized through Bayesian framework and evidence theory [40]. Therefore, the association between static and dynamic perception results is realized.

Association of dynamic targets and static environment makes full use of the motion information provided by MMW radar and plays a significant role In autonomous driving.

## 3. MMW Radar Perception Approaches

MMW radar plays an important role In the driving assistance and autonomous driving. According to MMW radar’s low cost and robust working conditions, it has been widely applied on Levels 1 and 2 of driving automation defined by SAE (Society of Automotive Engineers) [4] and has already been used on production vehicles. Advanced Driver Assistance System (ADAS) belongs to active safety technology and is critical for L1 L2 vehicles. The object-layer data from radar can provide frontal objects’ position and speed, which is key information to ADAS to detect and track dynamic and static obstacles [5]. In ADAS functions such as Frontal Collision Warning (FCW) [41], Lane Change Warning (LCW), and Autonomous Emergency Braking (AEB), these perception results help the system to timely find and avoid driving risks that vehicles may encounter. Moreover, the tracking results which come from radar data provide the preceding vehicle’s relative motion information. These can be used to control the self-vehicle’s longitudinal and lateral dynamics and maintaIn a safe distance from the preceding vehicle, which reduces driver fatigue In adaptive cruise control (ACC) [6]. Because MMW radar can adapt to different weather conditions, and it is the only sensor that can directly measure objects’ speed for a long range at present, MMW radar cannot be replaced by other sensors for the time being.

For higher-level automated vehicles, though the maIn perception schemes tend to choose LIDAR and vision sensors as they acquire richer and more precise information, MMW radar is a significant supplement of information source to LIDAR and cameras both In adverse weather conditions and blind areas.

Generally speaking, radar-based perception approaches contaIn two parts. On the one hand, radar information has been studied for a long time to realize object detection and tracking. Radar usually cooperates with other sensors to improve the detection results. With the development of deep learning, new methods are used to process radar data and improve the precision and accuracy. On the other hand, other research works use radar to realize self-localization of vehicles after static environment mapping. In addition, recently some map manufacturers claimed that radar-based HAD map has been used to support automated driving.

### 3.1. Object Detection and Tracking

#### 3.1.1. Radar-Only

In recent years, more and more studies employ diversiform methods to enhance the results of object detection and classification based on MMW radar data [23,24,42]. Researchers chose to process radar data with neural networks or grid-mapping to obtain rich target perception information.

Because the MMW radar point cloud is relatively sparse and objective characteristics are not obvious, using DL methods to realize object detection and classification is very challenging based on this type of data. According to related research works, there are mainly two approaches to solve this problem at present. The first method is using radar-based grid maps determined by the accumulation of multiple frames data. As gridmaps are not quite sparse, they can improve this problem to a certain degree. Then use segmentation networks to process radar-based gridmaps like processing images [15,42,43]. Connected area analysis and convolutional neural network are used in [15]. Then radar grid map can be used to classify static traffic targets (vehicles, buildings, fences) and recognize different orientations of targets represented in grids. Furthermore, the full convolutional neural network (FCN) [44] is used to conduct semantic segmentation for radar OGM, to distinguish vehicles and background information in OGM at pixel level [43]. In the newest research, occupancy grid map, SNR grid map, and height grid map constructed from high-resolution radar are regarded as three different channels, which are sent to semantic segmentation neural network FCN, U-Net [45], SegNet [20], etc., for the segmentation and classification of multiple traffic targets in the environment [42].

**Figure 6:**
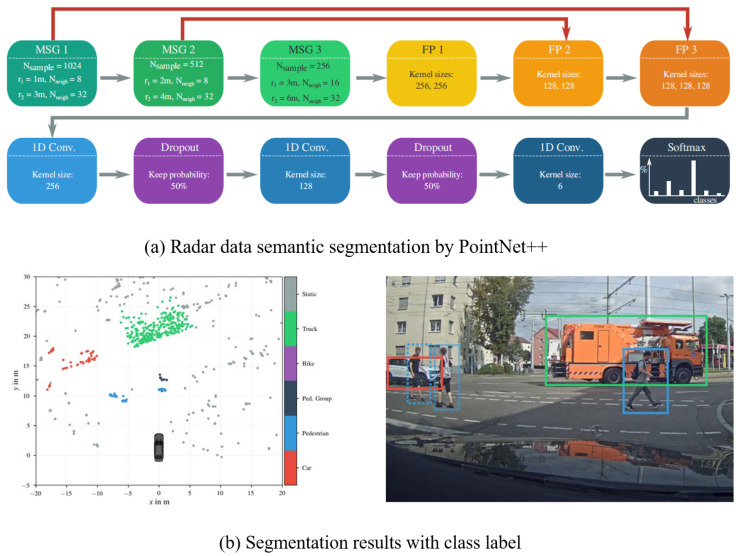
Semantic segmentation on radar point cloud [23].

However, the segmentation network based on grid maps can only be used to classify static targets and cannot be fully used for the dynamic detection capability of MMW radar. Therefore, In other research works, the second method using DL to process radar data is directly processing the original radar point cloud as LIDAR data is more similar to radar data than images. Furthermore, these studies modify the network to make it more suitable to the density and sampling rate of radar data [23]. In [23], it is mentioned that the modified neural network PointNet++ [21] is used to sample and cluster the radar measurement, while a semantic segmentation network is used to obtain the point cloud level classification results. The data processing flow and segmentation result are displayed in Figure 6. However, the shortcoming is that the detection outputs are not integrated at the object level. In [46], a 2D target vector table determined by radar is used to represent targets around a vehicle and perception accuracy, so as to further detect the parking space adjacent to the vehicle. Besides CNN, RNN network LTSM (Long–Short-Term Memory) is used to classify pedestrians, vehicles, and other traffic targets in [24,47], and can identify categories that have not been seen during the training.

#### 3.1.2. Sensor Fusion

Object detection and classification is a key aspect of environment perception where MMW radar plays an important role. Complex and dynamic traffic environment requires high accuracy and strong real-time performance of the vehicle perception system [48], especially in highly automated driving. As sensor fusion complements sensors’ advantages to improve the accuracy, real-time performance, and robustness of perception results, plenty of research works focus on multi-sensor information fusion. MMW radar is a common autonomous sensor used for multi-sensor fusion in object detection and tracking.

One common sensor fusion detection solution is combining MMW radar and visual information. It takes advantage of rich semantic information from images as well as position and movement information from radar to improve the confidence of perception results, obtain more detailed environmental information, and build a good foundation for decision-making and control of intelligent vehicles [49]. Radar-vision fusion is mainly divided into data-level fusion and object-level fusion.

**Figure 7:**
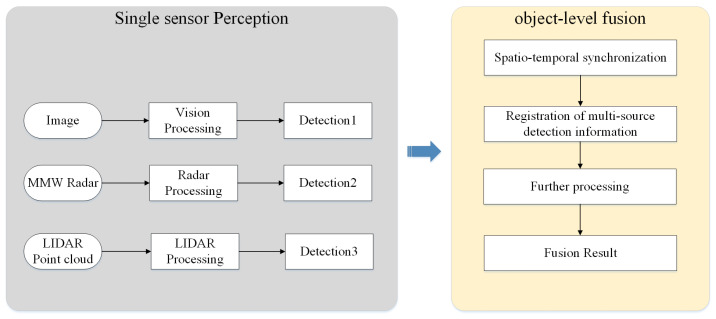
Overview of object-level fusion.

For object-level fusion, at first each sensor processes raw measurement separately. For radar, single-sensor data processing is mainly carried out from the perspective of kinematics. For visual data, studies usually adopt machine learning methods to extract Haar features or Hog features [50] and use SVM or Adaboost to identify specific categories of objects [51]. With the development of deep learning, Faster RCNN [22], YOLO [52], and SSD [53] predict object bounding boxes and classification jointly with outstanding accuracy. Therefore, more and more object-level fusion algorithms use deep learning methods for image processing. The perception results of single sensors are then matched and fused to determine the final result [54] to improve the detection confidence and accuracy [55] and realize joint tracking at further steps [56]. Data association is needed to match perception results of different single sensors. Frequently used algorithms for data association include the nearest-neighbor algorithm (NN) [57], probabilistic data association such as joint probabilistic data association (JPDA) [58], and multiple hypothesis tracking (MHT) [59]. Then state filters such as Kalman Filters (KF) [60], Extended Kalman Filters (EKF) [61], and Unscented Kalman Filters (UKF) [62] are commonly applied to solve the problem of multi-sensor multiple object tracking. Bayesian probabilistic reasoning method and the Dempster-Shafer (D-S) theory of evidence [63] are often used to cope with uncertainty and conflicts on detection results from different sensors [64]. Figure 7 shows the overview of object-level fusion. Moreover, these fusion theories are also used for hybrid-level fusion and proved to be effective when tested on real data [65]. In conclusion, object-level fusion framework has a small dependence on single sensor and is robust to single-sensor failure. However, it also has obvious information loss, and fails to take full advantage of sensor data [66].

For data-level fusion, the raw information of all sensors is transmitted to a fusion center for centralized data processing. Through joint calibration, the conversion between the spatial relation of the two sensors is established. Radar provides the Region of Interest (ROI) which indicates an object’s location. Then ROIs are projected onto the image space [67]. Then, deep learning [68] or machine learning [69] are used to realize visual object detection and classification. Data-level fusion makes image processing more targeted and improve the algorithm’s efficiency [70]. However, if radar information contains numerous false detections or missed detections, the accuracy of data-level fusion results will be impacted greatly [71]. Moreover, data-level fusion requires high accuracy of spatio-temporal correspondence of multiple sensors and high communication bandwidth [72]. Therefore, the computing load of the centralized fusion center is large, which poses a challenge to the real-time perception. With the development of DL, vision detection algorithms using CNN have achieved excellent performance on the accuracy and efficiency at the same time. The main advantages of classical data-level fusion are gradually replaced, so the subsequent research using machine learning are gradually reduced by deep fusion.

**Figure 8:**
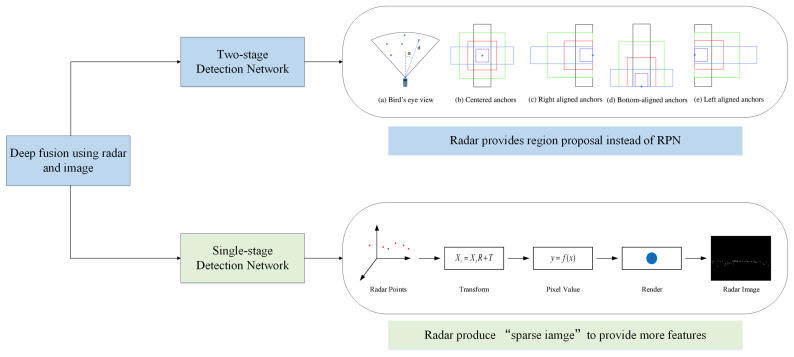
DL architectures on radar-image fusion [26,73].

With the development of deep learning and neural networks, deep fusion has become the latest trend of radar-vision fusion. According to different implementation methods, deep fusion of MWR and vision can be divided into two kinds. The first one regards the image coordinate system as the reference coordinate system. According to different object detection frameworks, deep fusion can be divided into two-stage detection [73] and one-stage detection [74]. Figure 8 shows the radar processing procedure of this two kinds of deep fusion.

In the two-stage detection, the position of objects provided by radar replaces the role of region proposal network (RPN), and image information is further used to realize the refinement of the candidate area and the classification of objects. In addition, the related algorithm using RPN with Fast RCNN has been proved to be more efficient and accurate than the same backbone with selective search [73].

In single-stage detection, YOLO, SSD, and other networks are used to solve the unified regression problem of object location and classification. Compared with the two-stage target detection network, single-stage detection is faster, but the accuracy is lower. The detection performance can be further improved by integrating MMW radar information. Related networks commonly receive the input of image and radar data, respectively. The radar information is then converted into image format information, which is used as training data with the images together. The deep fusion neural network will learn to fuse different features to realize better performance. The key to these algorithms includes the following parts. At first, it is significant to design the rule of generating a “radar sparse image”. These algorithms project radar data to image coordinate system. Moreover, as it is known that radar data is sparse compared with image, these algorithms try to make the best of different dimension information of radar measurement, such as distance, speed, and intensity to fill multiple channels of “radar sparse image” [26]. Multi-frame data is also used here to increase the density of radar data [25]. Secondly, to ensure at what level the fusion of radar and image data is the most beneficial, feature pyramid networks (FPN) is applied [25]. Thirdly to address the unbalance between positive and negative samples, focal loss is adopted to design the loss function. [26,75]. Under multifaceted efforts, deep fusion networks reveal their good performance as is showed In Table 2.

According to the experimental results on nuScenes such as CRF-Net [25], RVNet [74], and SAF-FCOS [26], deep fusion can surely improve the detection results of vision detection, especially In adverse weather conditions and nights or for small-size objects detection.

Moreover, the sensor fusion between LIDAR and MMW radar can be used to further improve the estimation of objects’ semantic information and dimension. Both sensors can provide location information of objects. As for complementary information, LIDAR provides high-resolution information about object contours, while radar provides Doppler velocity information. The tracking of extended dynamic objects becomes more reliable and robust under sensor fusion [78,79,80].

At present, studies on the fusion of millimeter-wave radar and other sensors with DL neural network have also made some progress [25,26], which will also be an important research direction of multi-source sensor fusion perception in the era of artificial intelligence.

### 3.2. Radar-Based Vehicle Self-Localization

For highly automated driving, accurate pose (i.e., position and orientation) estimation in a highly dynamic environment is essential but challenging. Autonomous vehicles commonly rely on satellite-based localization systems to localize globally when driving. However, in some special situations such as near tall buildings or inside tunnels, signal shielding may occur which disturbs satellite visibility. An important compensation method to realize vehicle localization is based on environmental sensing. When a vehicle is driving, sensors record distinctive features along the road called landmarks. These landmarks are stored in a public database, and accurate pose information is obtained through highly precise reference measuring. When a vehicle drives along the same road again, the same sensor builds a local environmental map and extract features from the map. These features are then associated with landmarks and help to estimate the vehicle pose regarding landmarks. The vehicle’s global pose is deduced from the landmarks’ accurate reference pose information. Technologies used in this process include the sensor perception algorithm, environmental map construction, and self-vehicle pose estimation. Meanwhile, as the driving environment changes with time, environment mapping also needs the support of map updating technology [81].

**Figure 9:**
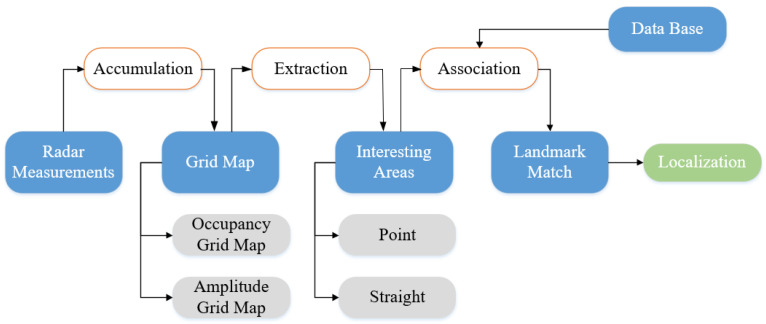
Overview of radar-based vehicle localization.

To realize vehicle localization and map updating, different mapping methods are used with different sensors. For ranging sensors, the LIDAR is typically used to represent environmental information in related algorithms due to its high resolution and precision. For vision sensors, feature-based spatial representation methods such as the vector map are usually established which take less memory but more computational cost than the former. Compared with LIDAR and camera, radar-based localization algorithms are less popular because data semantic features provided by radar are not obvious and the point cloud is relatively sparse [82]. Nevertheless, recent research works begin to attach importance to radar-based vehicle self-localization [83,84]. Since radar sensors are indifferent to changing weather, inexpensive, capable of detection through penetrability, and can also provide characteristic information needed by environmental mapping and localization [3]. Thus, radar-based localization is a reliable complementary methods of other localization techniques and the research work is challenging but meaningful [85]. Through multiple radar measurements, a static environment map can be established, and interesting areas can be extracted according to different map types. Then these areas can be matched with landmarks which have been stored in the public database. Finally, the vehicle localization result can be obtained through pose estimation. This process is illustrated in Figure 9. According to the distinction of mapping methods, radar-based localization algorithms are often presented in three kinds: OGM, AGM, and point cloud map. In addition, according to the different map data formats, different association methods and estimation methods can be applied. For OGM, classical SLAM algorithms which use state filters such as EKF and PF are often chosen to realize further data processing. Or we can regard OGM as an intermediate model for features-based spatial expression and combine graph-SLAM with OGM to accomplish feature matching. While using AGM as the map representation, algorithms such as Rough-Cough are applied to match interesting areas with landmarks. As to point cloud map, Cluster-SLAM is proposed to realize localization. The distinctions between these methods are listed in Table 3.

Different radar-based mapping algorithms of the current local static environment influence the quality of available distinguishable features. This is the key point to match the landmark exactly. Regarding the proceeding positioning algorithms, In the early stage, classical SLAM algorithms were used to realize self-localization by using EKF with radar data features [86], or by using sensor modeling and sequential Monte Carlo (particle) filtering algorithms [87]. The proceeding content is organized according to different radar-based map forms applied to vehicle self-localization.

The most direct method to realize radar-based self-localization is building OGMs and extracting the relevant interested environmental information from OGMs. However, this method is only suitable to establish a static localization system. Some scholars adjust the measurement model to make it adaptable for dynamic detection [88] or track dynamic targets In the process of map construction [89]. These methods only improve the localization of short-term dynamic environment. In [17], through random analysis of interesting area from prior measurements and the semi-Markov chain theory, multiple measurements based on OGMs are unified to the same framework. This approach can improve localization effect when the environment is in long-term change, but still cannot solve the problem of complete SLAM.

To decrease memory cost, OGMs can also be used as an intermediate model for features-based spatial expression. Grid-based expression is constructed for local observation environment, and independent feature information was extracted from it in [90]. In [91], the authors use the feature information determined from OGMs and graph-SLAM to realize vehicle localization. In [92], they use graph optimization to solve the SLAM problem on optimized maps. They extract feature information through the local OGMs constructed around the vehicle and use a SLAM-related algorithm to obtain pose estimation and map optimization. They realize localization problems based on these results. However, feature-based localization algorithms relied heavily on the extraction of suitable features. These mentioned algorithms were only evaluated on small-scale datasets collected in a parking lot, and whether they are efficient enough for lane-level localization was not verified.

**Figure 10:**
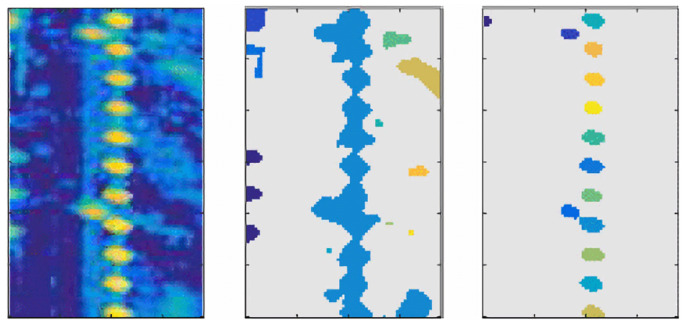
Interesting areas extracted by AGM for localization [18].

In addition to the use of OGMs to describe the environment, MMW radar data can also be used to build AGMs to achieve vehicle localization and map updating. AGMs can distinguish metal, roads, and vegetation, which has its own unique advantages. Researchers in [18] mention two ways to express interesting areas In AGMs: point-shaped areas and straight areas. The characteristics of interesting areas can be extracted through DBSCAN, MSER, or connected region, as shown in Figure 10. An online recognition and registration method known as Rough-Cough is proposed for extracting features from AGMs in [93]. This method does not require input images with very clear structures and is suitable for all image feature pairs that can be aligned through Euclidean transformation with low mismatch rate and registration error. The key point of related algorithms is the correlation effect between features and landmarks. Straight features are favored because of the obviously larger size compared to point features. By measuring the distance between straight segments effectively, feature information can be correlated and matched with the database [94], and performance of different algorithms have been already evaluated in [95]. Moreover, new progress has also been made in the correlation method of points-shaped interesting areas with landmarks [96].

**Figure 11:**
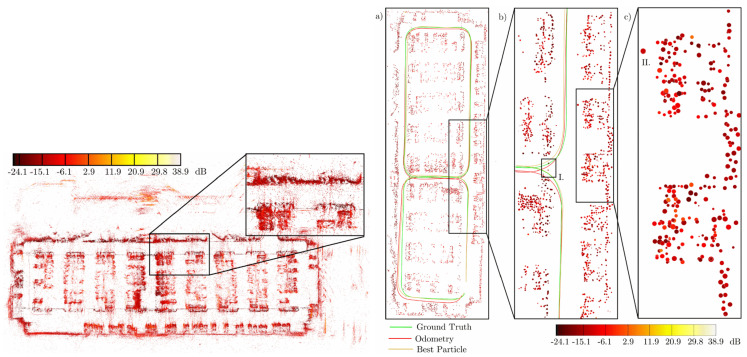
An illustration of Cluster-SLAM mapping [19].

Besides the methods mentioned above, another method known as Cluster-SLAM represents environment information differently as is shown in Figure 11. It integrates radar data into multiple robust observations using the stream clustering method. In addition, then it uses the particle filter to achieve map matching and pose estimation [19]. The expression of data in this method is similar to the expression of feature space extracted from radar grid maps. Using a FastSLAM algorithm for map construction and pose estimation has been proven to be a feasible scheme. However, it also has some disadvantages. It is difficult to adjust parameters of the particle filter to determine clustering radius in the actual situation. As the actual situation is complex and time-varying, obtaining a suitable clustering radius which is a crucial factor to the map representation is hard. The number of particles in PF may increase to a large number. In addition, this will bring about an increasing of computational burden.

Apart from the type of mapping method, sufficient localization accuracy for high-level AVs is crucial for safe driving. Decimeter-level or even centimeter-level accuracy is required, and real-time efficiency should be considered simultaneously. Up to now, some promising results of radar-based localization have been acquired. In the work of [97], based on the matching results of environmental features and landmarks detected by radar, the iterative closest point (ICP) algorithm and EKF are used to realize positioning, which can give consideration to accuracy and algorithm complexity. The RMS errors of results is 7.3 cm laterally and 37.7 cm longitudinally. In [83], the study used RTK-GNSS and radar to realize static environment map generation and localization by modeling uncertainties of sensors. The longitudinal and lateral RMS errors is around 25 cm. These results show a promising prospect to apply radar-based localization algorithms on AVs.

According to the above review, radar-based self-localization has been proved to be feasible for AVs. Low economic cost and memory makes relevant algorithms suitable for the production of high-level AVs. Moreover, radar-based positioning can be available for AV localization in bad weather, when sensors such as LIDAR and cameras perform poorly. Therefore, new studies which focus on radar-based SLAM are trying creative methods to make up for the defects of radar data [84,98].

## 4. Future Trends for Radar-Based Technology

Through the above review of MMW radar-based technologies applied in autonomous driving, we reach the following conclusion.

**Figure 12:**
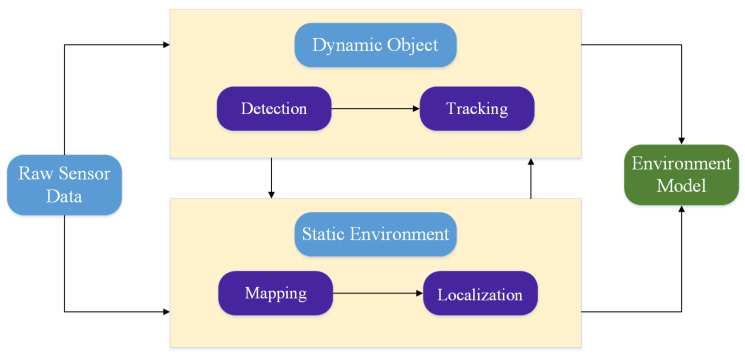
Overview of environmental perception tasks for autonomous driving.

1. **MMW radar is widely used in perception tasks for autonomous driving.** We divide environmental perception tasks towards two types as is shown in Figure 12. For dynamic objects, object detection and tracking can be employed to obtain objects’ position, motion, dimension, orientation, and category etc. For static environment, through SLAM we can get the environmental mapping information and determine the pose of the self-driving vehicle. In the past and present, MMW radar plays an important role in all these tasks. It cannot be replaced by other sensors to the ground. Therefore, studies about MMW radar-based environmental perception algorithm are important.
2. **Multi-sensor fusion and DL attracts a lot of attention and become increasingly significant for radar-related studies.** As fusion combines advantages from different sensors and improve the confidence of single-sensor data processing result, it is a good choice to fuse radar data with others. Radar can provide measurement of speed and other sensors can provide semantic or dimensional information. Moreover, fusion can surely offset against the low resolution of radar data. Radar-related fusion studies include data-level fusion, object-level fusion. In addition, with the release of dataset for autonomous driving which provide radar data, more and more researchers pay attention to train radar data with DNN. Some works which use radar data solely or deep fusion have obtained good results on detection, classification, semantic segmentation and grid-mapping. Although current networks used to process radar data are usually modified from NN used to process image and LIDAR point cloud, we believe with the revealing of more essential characteristics to describe object features, there will be more progress about radar-based deep learning algorithms.

Although the research works related to MMW radar in AVs have attracted plenty of attention, the following requirements are necessary for sustaining improvement.

1. **Improvement of radar data quality:** Many studies prove that when using radar data, it is difficult to eliminate noise from radar data in both tracking [65] and localization tasks [92]. Therefore, the way to enhance the anti-jamming ability of radar to clutter is a challenge that cannot be ignored.
2. **More dense and various data:** In many research works, we find that the main limitation of MMW radar-based algorithms is in its sparse data which is hard to extract effective features. Compared with LIDAR, the lack of height information also restricts radar’s use in highly automated driving. Adding three-dimensional information to radar data can surely contribute to automotive radar’s application [31]. Therefore, the MMW radar imaging ability must be further improved, especially with regards to the angular resolution and increase in height information.
3. **More sufficient information fusion:** Because the perception performance and field of view (FOV) of a single radar is limited, to improve the effect and avoid blind spots, information fusion is necessary [99]. Fused with information of vision, high automated map [100] and connected information [101] will enhance the completeness and the accuracy of radar-based perception tasks, which improve safety and reliability of autonomous driving ultimately. in the process of fusion, how to obtain precise time-space synchronization between multi-sensors, how to realize effective data association between heterogeneous data and how to obtain more meaningful information by fusion deserves careful consideration and more academic exploration.
4. **Introduction of advanced environmental perception algorithm:** Deep learning and pattern recognition should be further introduced in radar data processing, which is important to fully excavate the data characteristics of radar [2]. How to train radar data with DNN effectively is a problem in urgent need of a solution.

## 5. Conclusions

In summary, in the face of dynamic driving environment and complex weather conditions, MMW radar is an irreplaceable selection among the commonly used autonomous perception sensors. in the field of autonomous driving, many modeling and expressions from radar data have been realized. In addition, various applications or studies have been realized in the fields of active safety, detection and tracking, vehicle self-localization, and HD map updating.

Due to the low resolution and the lack of semantic features, radar-related technologies for object detection and map updating is still insufficient compared with other perception sensors in high autonomous driving. However, radar-based research works have been increasing due to the irreplaceable advantage of the radar sensor. Improving the quality and imaging capability of MMW radar data as well as exploring the radar sensors’ use potentiality makes considerable sense if we wish to get full understanding of the driving environment.

## Figures and Tables

**Table 1:** Model analysis based on MWR data.

Task	Data Format	Algorithm	Advantages and Usefulness	Ref.
Dynamic Targets Modeling	Cluster-layer data	Estimation extended objects by Doppler effect	1. Estimate the full 2D motion of extended objects; 2. Used to track dynamic extended object	[9,10,29]
Dynamic Targets Modeling	Cluster-layer data	Clustering based on DBSCAN	1. Estimate the dimension of extended objects	[7,8,30]
Dynamic Targets Modeling	R-D Map	Frequency spectrum analysis	1. ObtaIn the category of dynamic objects	[11,12]
Static Environment Modeling	Cluster-layer data	Occupancy grid maps	1. Used to realized road scene understanding and localization	[13,14,31,32]
Static Environment Modeling	Cluster-layer data	Amplitude grid maps	1. Reflect the characteristics of objects besides environmental mapping	[13]
Static Environment Modeling	Cluster-layer data	Free Space	1. Display of available driving areas Valuable to vehicle trajectory planning	[33,34]

**Table 2:** The performance of Radar-Camera Deep Fusion.

Algorithm	Baseline	Performance on nuScenes [27]	Improvement
SAF-FCOS [26]	FCOS [76]	mAP 72.4%	mAP 7.7%
RVNet [74]	TinyYOLOv3 [77]	mAP 56%	mAP 16%
CRF-Net [25]	RetinaNet [75]	mAP 55.99%	mAP 12.96%

**Table 3:** Analysis of radar-based self-localization methods.

Method	Strengths	Shortcomings
Occupancy Grid Map	Most common algorithms used in radar-based SLAM	Require lots of computation cost when updating map
Amplitude Grid Map	Distinguish different materials according to reflection characteristics	Less clear position representation compared to OGMs
Point cloud Map	A robust and efficient mapping method saving lots of time and memory	Difficulty of adjusting parameters of particle filter

## Abbreviations

The following abbreviations are used In this manuscript:

MMW	Millimeter Wave
AV	Automated Vehicles
ADAS	Advanced Driving-Assistance Systems
DL	Deep Learning
OGM	Occupancy Grid Map
AGM	Amplitude Grid Map
SLAM	Simultaneous Localization And Mapping
